# Long term outcome study of a salutogenic psychoeducational recovery oriented intervention (Inte.G.R.O.) in severe mental illness patients

**DOI:** 10.1186/s12888-022-03887-2

**Published:** 2022-04-05

**Authors:** Franco Veltro, Gianmarco Latte, Irene Pontarelli, Cristina Pontarelli, Ilenia Nicchiniello, Lilia Zappone

**Affiliations:** Department of Mental Health – ASReM, Campobasso, Italy

**Keywords:** Psychoeducation, Salutogenesis, Recovery, Group intervention, Long-term follow-up, Public health

## Abstract

**Aim:**

Inte.G.R.O. is a standardized Salutogenic-Psychoeducational intervention designed to help people with severe mental illness manage their life-stress and achieve personal recovery goals through the improvement of social functioning. The aim of this study is to evaluate the long-term outcome of this approach, characterized by health promotion rather than correction of dysfunctional strategies.

**Methods:**

41 people underwent an *observational study* with a three time-point evaluation (t0, pre- treatment; t1, 12 months; t2, 36 months). At each time point, social functioning was assessed as primary outcome by the Personal and Social Functioning scale (PSP); furthermore, psychopathological status was assessed by Brief Psychiatric Rating Scale (BPRS), stress management was measured by means of Stress-Scale and cognitive flexibility variables were assessed by Modified Five-Point Test (M-FPT).

**Results:**

Personal and Social Functioning increased at t1 and t2 vs t0; psychopathological status improved at t2 vs t0; stress management improved at t2 vs t1; cognitive flexibility improved at t2 vs t0.

**Conclusions:**

these results substantially confirm after a three-year follow-up the improvements in functioning, psychopathology, stress management and cognitive flexibility seen in previous studies. Furthermore, they show a complex time-dependent fashion. Overall, they confirm a remarkable and long-term impact of Inte.G.R.O. on key Recovery variables. Further studies are needed to address extent and duration of these improvements.

**Supplementary Information:**

The online version contains supplementary material available at 10.1186/s12888-022-03887-2.

## Introduction

One of the best definitions of Recovery in Severe Mental Illness (SMI) belongs to SAMHSA [1] “a process of change through which individuals improve their health and wellness, live a self-directed life, and strive to reach their full potential”. We share the concept that recovery is a journey characterized by the commitment of the user to become more and more capable and responsible in his life. This condition could be achieved by acquiring life skills; then, improving the needed abilities is the aim of a recovery-oriented approach much more than focusing on the treatment of the symptoms, on the deficit and so on. According to Mueser [2], the argument that recovery from severe mental illness “should define in terms of improved functioning and establishing a rewarding and meaningful life, rather than the permanent remission of symptoms and impairments, has gained traction since it was proposed more than 20 years ago”.

From this point of view, it is obvious that the milestone concept is the improving of functioning [3], often used in the specific literature as primary outcome [4, 5], to assure the independence with a minimum support by professionals. That means a secure base to facilitate the recovery.

In the recovery process the definition of SMI in not defined with operational criteria, and the most shared and simple definition refers to people with psychological/psychiatric problems *often* so debilitating that one’s ability to engage in functional and occupational activities is severely impaired. On this premise Inte.G.R.O. has been developed to support people with SMI in their recovery process [6], promoting resilience as a dynamic process of positive adaptation in the face of adversity [7] obtained by a continuous amelioration of functioning. Consistently with this concept, Inte.G.R.O is aimed at promoting Personal Goal Definition, Problem Solving, Emotional literacy, emotional perception, abilities to accept adverse conditions, communication skills, assertiveness included. All these components belong more to a salutogenic approach than to a “reparative process” as known in traditional rehabilitative treatment; no advice is given for coping with symptoms or with function deficits. We believe that the approach characterized by salutogenesis in terms of Antonovsky’s theory [8–10] and psycho-education as structured by Falloon [11–13] is the best innovative way to improve functioning in order to facilitate recovery. Antonovsky’s theory and pychoeducation as conceived by Falloon share themes such as stress theory, coping with stress, health/life, problem solving. This approach is also consistent with Antonovsky’s theory, stating that stress is a challenge that requires personal commitment.

Inte.G.R.O is a structured and manualized group approach. Its manual contains written questions and possible users’ answers to be rapidly discussed. It includes the modules illustrated in Appendix 1. Inte.G.R.O, coherently with its scope, has been conceived only for users in a phase of relative clinical stabilization, close to the concept of clinical remission [14], with relative awareness of the disorder, with a functioning score over 40 at the Semi-Structured Interview “Personal and Social Performance Scale” (PSP), [15] without any severe cognitive deficits that may compromise learning and group participation. To date Inte.G.R.O is limited among the Severe Mental Illness to patients within the diagnostic spectrum of schizophrenia, bipolar and personality disorders regardless of the diagnostic subtypes.

As generally known, there are several approaches to facilitate recovery. The most used are Refocus [16], also in the version for General Practitioner [17], Wrap [18], E-IMR [3] and recently ART, a new approach proposed by Zomer J. C. et al. [19]. Refocus and ART share many principles and strategies. Refocus, as known, is used to train professionals to address services towards recovery-oriented processes. Undoubtedly these are interesting approaches, useful and complementary with rehabilitation treatments or with other functioning-improving practices, but as for the methodologies and strategies are not comparable with Inte.G.R.O, which is based on users’ group work. Wrap focuses attention on the illness management, with the primary outcome on the symptomatology, and for this reason is very different from Inte.G.R.O, which as we underlined does not work with symptom management or “deficit-reparative” treatments. We found Illness Management and Recovery [3] the most similar approach to Inte.G.R.O as for the following aspects: defining goals, problem-solving, stress-management, communication, life-style. The difference concerns only the aspect of illness management but not the Problem Solving approach used in E-IMR. Indeed, Problem Solving is applied to practical, interpersonal and personal problems with Inte.G.R.O, since we believe it is fundamental for the recovery journey. Nevertheless, Problem Solving is generally underestimated, although in previous studies good results were obtained about its application to social functioning [20–23].

The aim of this study is to investigate the long-term effect of Inte.G.R.O using improvement of social functioning as primary outcome. We evaluated the level of stress, cognitive flexibility, clinical symptoms and admissions in psychiatric ward as secondary outcomes.

## MATERIALS and METHODS

### Study design

This is an observational study without a control group with a long-term follow-up. In fact, a three time-point evaluation (pre- treatment, post-treatment-12 months and 36 months after the beginning of the treatment) was carried out on personal and social functioning, psychopathological status, stress management and cognitive flexibility variables. The patients were asked written consent to attend the course for a year at least, to be evaluated by several questionnaires and interviews, for the collection of data for research purposes.

### Sample

A total of 41 users of the Mental Health Service (MHS) who participated to the Inte.G.R.O training were evaluated. The catchment area of MHS is approximately 95.000 adult inhabitants, of which ¾ in urban area with no high population density and ¼ in rural area. The accessibility of MHS is good. It is important to consider that the participation to the training was conditioned by the following factors, that are consistent with the scope and the characteristic of this approach: the first was the determination of the patients to undertake a journey towards recovery; the second was that they had attended an individual and /or familiar and/or group psychoeducational course; the third was social functioning greater than or equal to 40 as assessed by the PSP scale. On the basis of these characteristics, according to the spectrum diagnostic criteria of schizophrenia, bipolar or personality disorder, all the psychiatrists or psychologists gave the patients information about Inte.G.R.O approach and asked if they were willing to participate in the training. As a consequence, as for this observational study, for all patients the consistent inclusion criteria were a diagnosis of Schizophrenia, Bipolar disorder or Personality disorders, functioning ≥40 on the PSP scale, age 18–65. Exclusion criteria were mental retardation and substance abuse.

### Specific and technical characteristics of Inte.G.R.O

The maieutic attitude and socratic questioning [24] (Perris, Blackburn & Perris, 1988), the stimulation of peer-to-peer and cooperative learning, together with the “Group Cohesiveness”, represent Inte.G.R.O. technical characteristics. The intervention requires two trained professionals, a conductor and a co-conductor, competent in psychoeducational principles and mastering basic cognitive-behavioral techniques. The conductor and co-conductor must use the manual, where all steps are described in detail for each meeting, sharing specific written sections of it with participants. However, the first part of the manual illustrates for the professionals: the theoretical principles, the basic cognitive-behavioral techniques, the conduction style, the tools to enhance skills and to monitor improvements. The second part contains the scripts of all sessions/meetings of the program. There are usually 36 weekly meetings, each one lasting 90 min (Appendix 1); some of them are carried out twice a week to facilitate learning on the subject. The reinforcement and supervision sessions are held every 15 days, then monthly and finally every 3 months. Therefore, a booster session is offered once a month for 3 months, once in a three-month period and the last session after 6 months, for a total of 5 meetings in 1 year. The intervention is based on four fundamental modules (definition of Pleasant and Personal Goals, effective communication, emotional perception and problem-solving), each comprising different teaching units. The most used module is that of Problem Solving, also extensively applied during the training. For each unit a meeting is scheduled. There are also intermodular educational units to facilitate the acquisition of the skills of single modules (Appendix 1). Every single session begins with the revision of the previously assigned tasks, with an emotional roll call and with brief body exercises; after that, the topic of the teaching unit is discussed, by means of role-playing if indicated. The last phase concerns homework assignment. A constant monitoring and support for the pleasant/personal goals chosen and defined by each participant are carried out.

### Assessments

The assessment of the primary outcome was performed by means of the Italian version of Personal and Social Functioning scale [15]. The PSP evaluates personal and social functioning through a semi-structured interview and the information available from the acquaintances and health workers themselves. There are four main areas: 1) socially useful activities (including working and studying) 2) personal and social relationships 3) taking care of appearance and hygiene 4) disturbing and aggressive behaviours. The total score ranges from 0 (worst possible functioning) to 100 (excellent functioning).

Secondary outcome measures were evaluated by means of: BPRS [25], Stress Scale [26], Modified Five-Point Test [27]. The Italian version of BPRS [28, 29] is a psychopathological hetero-evaluation scale consisting of 24 items. Each item is assessed on a Likert scale with 7 coding levels ranging from 1 (absent) to 7 (very severe). The Stress Scale^30^ is made up of 9 items taken from the well-known and widespread Goldberg tool for investigations in routine conditions with a dichotomous yes-no answer which evaluates the presence of stress if the score is greater than 5. The M-FPT in its Italian version [27] is a test for measuring non-verbal fluency (figurative) of executive functioning, linked to cognitive flexibility. The subject is asked to draw different figures (images) in some minutes, following some rules and avoiding repetitions. The main aspects assessed are: cognitive flexibility, presence or absence of perseveration and strategic performance. The assigned scores are of 3 types: 1) Unique Drawings (UDs), i.e. number of drawings valid according to the rules and not produced before; 2) Cumulative strategies (CSs) i.e. number of UDs produced with a particular strategy that can be either enumerating (CSse) or rotative (CSsr) strategy; 3) Error index (Errl), i.e. a percentage between the number of perseverative drawings or breaking rules (errors) and the total number of drawings.

### Statistical analysis

For all the variables with parametric distribution the average ± sd was calculated; for all variables with non-parametric distribution the median and range were calculated. A three time-point evaluation of the variables was performed. Normally distributed variables were analyzed by means of parametric tests, such as one-way Repeated Measures ANOVA, with subsequent pairwise comparisons. We additionally performed post-hoc Bonferroni-corrected paired t-tests. Non-normally distributed variables were analyzed by means of nonparametric tests, such as a Friedman test, or Cochran’s Q test with post-hoc Bonferroni-corrected McNemar test, with post-hoc Bonferroni-corrected Wilcoxon tests. SPSS 21.0 software (SPSS Inc., Chicago, IL, USA) for macOS was used for statistical analysis.

All methods were carried out in accordance with relevant guidelines and regulations. The need of ethical approval and informed consent was exempted by ASReM local ethical committee N 950/21.

## Results

### The socio-demographic characteristics of the sample and the statistical results are shown in Tab 1

Among the 41 subjects selected, 24 had a diagnosis of psychotic disorder; 10 had a diagnosis of mood disorder; 7 had a diagnosis of personality disorder. Among subjects with a diagnosis of psychotic disorder, 19 were diagnosed with Schizophrenia, 2 with Schizoaffective Disorder (bipolar type), 2 with Delusional Disorder, 1 with Other Specified Schizophrenia Spectrum and Other Psychotic Disorder. Among subjects with a diagnosis of mood disorder, 7 were diagnosed with Bipolar I Disorder, 1 with Severe Major depressive disorder, 2 with Unspecified Bipolar and Related Disorder. Among subjects with a diagnosis of Personality Disorder, 2 were diagnosed with Obsessive-Compulsive Personality Disorder, 1 with Borderline Personality Disorder, 1 Histrionic Personality Disorder, 3 with Unspecified Personality Disorder.

**Table 1:** Socio-demographic characteristics of the Sample

Socio-demographic Characteristics			
*Gender*	Male (59%); Female (*41%*)		
*Diagnosis*	*Psychotic disorder (59%); Bipolar (24%); Personality disorder (17%)*		
*Relationship status*	***T0***	***T1***	***T2***
*Single*	*76%*	*73%*	*73%*
*Engagement*	*7%*	*10%*	*12%*
*Married*	*17%*	*17%*	*15%*
*Employment status*	***T0***	***T1***	***T2***
*Unemployed*	*83%*	*73%*	*71%*
*Employed*	*17%*	*27%*	*29%*

No drop-outs were observed. All patients regularly received drug therapy at T0, T1 (12 months) and T2 (36 months). No significative medication adjustments to optimize symptoms were observed since patients were clinically stabilized close to clinical remission criteria. The change of medication was relevant just for patients admitted to the psychiatric ward. However for all patients with personality disorders the main treatment was psychotherapy that was used for the maximum period of 18 months followed for three people by psychological support for 6 months. 5 out of 7 patients with bipolar diagnosis and 1 out of 2 patients with Schizoaffective disorders received weekly psychological support for the maximum period of 6 months. No patients attended occupational rehabilitation programs.

The mean age of subjects at the start of the intervention was 42.2 ± 10.6.

At T2 29 were “unemployed” (71%), 12 “employed” (29%). As we observed a trend toward significance at the Cochran’s Q test (*p* = 0.150), we decided to perform a post-hoc Bonferroni-corrected McNemar test, which resulted in: t0 vs t1, *p* = 0.063; t0 vs t2, *p* = 0.081; t1 vs t2, *p* = 0.246. Tested against a Bonferroni-adjusted alpha level of .016 (.05/3), no comparison remained significant.”

### The primary and secondary outcome of the sample and the statistical results are shown in Tab 2

During the year before treatment 2 patients (5%) had 2 “admissions” in psychiatric ward. During the active treatment (first year) 2 patients (5%) had 4 “admissions”. During the second and third year 4 patients (10%) had 5 admissions.

**Table 2:** Primary and secondary outcome

	m *±* ds			Pairwise post hoc		
	T0	T1	T2	T1 vs t0	T2 vs t0	T2 vs t1
*PSP*	*54.19 ± 10.62*	*59.19 ± 8.87*	*60.54 ± 9.01*	*T*_[_30_]_= − 7.52; p = <.001*	*T*_[_30_]_= − 5.94; p = <.001*	*T*_[_30_]_*= − 1.93; p = .060*
*Median (range)*						
*BPRS*	*43 (26–93)*	*38 (24–86)*	*35 (24–68)*	*Z = -2.12; p = .03*	Z = -2.91; p = .004*	*Z = -2.30; p = .02*
*Stress Scale*	*1.4 (1–2)*	*1.5 (1–2)*	*1.2 (1–1.9)*	*Z = .844 p = .399*	*Z = -2,39; p = .017*	Z = -3–42; p = −.001*
*M-FPT UDS*	*1 (0–4)*	*1 (0–4)*	*1 (0–4)*	*Z = -2.40; p = .016*	Z = -3.42; p = .001*	*Z = -1.58; p = .114*
*M-FPT CSs*	*1 (1–4)*	*3 (1–4)*	*2 (1–4)*	*Z = -2.50; p = .012*	*Z = -.704; p = .481*	*Z = -1.62; p = .104*
*M-FPT ErrIT*	*9 (0–64)*	*7 (0–40)*	*5 (0–54)*	*Z = -0.21; p = .829*	*Z = -2.15; p = .031*	*Z = -1.16; p = .245*

#### PSP

At t0 the average was 54.19 ± 10.62; at t2 it was 60.54 ± 9.01. Repeated-measures ANOVA: there was a significant effect of time, Wilk’s Lambda =0.41; F [2, 31]=27.62; *p* < .001. Three paired t-tests were used to make post hoc comparisons between time-points: there was a significant difference in the t1 vs t0 (*p* < 001) PSP score; and in the t2 vs t0 score (p < 001) but not in t2 vs t1 score.

#### BPRS

At t0 the median was 43.0 (range 26.0–93.0); at t2 it was 35 (range 24–68). A non-parametric Friedman test of differences among repeated measures was conducted and rendered a Chi-square value of 11.21 which was significant (*p* = .004). Post hoc Wilcoxon Signed-Ranks tests indicated that there was a significant difference in the t1 vs t0 score (*p* = .03), in the t2 vs t0 score (*p* = .004) and in the t2 vs t1 score (*p* = .02). Tested against a Bonferroni-adjusted alpha level of .016 (.05/3), only the t2 vs t0 comparison remained significant.

#### Stress scale

At t0 the median was 1.4 (range 1.0–2.0); at t2 it was 1.2 (range 1.0–1.9). A non-parametric Friedman test of differences among repeated measures rendered a Chi-square value of 12.10 which was significant (*p* = .002). Post hoc Wilcoxon Signed-Ranks tests indicated that there was a significant difference in the t2 vs t0 score (*p* = .017) and in the t2 vs t1 score (*p* = .001) but not in the t1 vs t0 score. Tested against a Bonferroni-adjusted alpha level of .016 (.05/3), only the t2 vs t1 comparison remained significant.

#### M-FPT

The UDs score showed a median of 1.0 and a range of 0.0–4.0 at t0, t1 and t2 (mean values: .97 at t0; 1.39 at t1; 1.63 at t2). A non-parametric Friedman test of differences among repeated measures rendered a Chi-square value of 13.97 which was significant (*p* = .001). Post hoc Wilcoxon Signed-Ranks tests indicated that there was a significant difference in the t1 vs t0 score (*p* = .016) and in t2 vs t0 score (*p* = .001), but not in the t2 vs t1 comparison. Tested against a Bonferroni-adjusted alpha level of .016 (.05/3), only the t2 vs t0 comparison remained significant.

At t0 the CSs median value was 1.0 (range 1.0–4.0); at t1 it was 3.0 (range 1.0–4.0); at t2 it was 2.0 (range 1.0–4.0). The Friedman test of differences among repeated measures showed no significant differences.

At t0 the ErrIT median value was 9.0 (range 0.0–64.0); at t1 it was 7.0 (range 0.0–40.0); at t2 it was 5.0 (range 0.0–54.0). The Friedman test of differences among repeated measures showed no significant differences.

## Discussion

The results of Inte.G.R.O intervention highlight a positive impact on the majority of outcome indicators. The trend of clinical crises was similar before and during the follow-up. Regarding the psychopathological component, it is worth recalling that the approach is not aimed at improving clinical symptoms, since it is addressed to patients with relative clinical stabilization similar to the concept of clinical remission by Andreasen et al. [14]. Neverthess, the post hoc comparison was significant for BPRS total score at T1 vs T0, and even more interestingly at T2 vs T1.

The greatest impact of the intervention remains, as shown in the two previous studies with a 12 month follow-up [6, 32], on the personal and social functioning, the primary outcome. The improvement of social functioning is impressive, ranging significantly from the average of 54.19 (±10.62), marked difficulties, at T0 to 60.54 (±9.01), evident difficulties (less severe) at T2. At T2 the percentage of unemployment decreased from 83 to 73%, with major change observed in the first period (t1 vs t0) with statistical probability not too far from significance (*p* = 0.06). We also have to consider that the context does not offer many job opportunities because of economic conditions.

Furthermore, qualitative data confirm these encouraging results. For example, one patient passed the competition for a position in the navy, 3 patients started working, one patient went to live alone, sharing the difficulties with some members of the group. Functioning of course is a prior target in recovery-oriented interventions [33] and the brilliant results concern a good social outcome associated with significant change in the real life.

In the previous studies we discussed about the role of Problem Solving in personal and social functioning since we evaluated after the active treatment the relationship between cognitive functions, in particular the planning activity and the Problem Solving [22]. A recent study observed that implementation of problem-solving strategies within Psychoeducational interventions may have an “impact on clinical and functional outcomes, by providing patients with long-lasting resources to manage daily life more effectively” [34]. Then, the surprising improvement in Personal and Social Functioning might be explained by the whole component of the approach as well as by the effect of Problem-Solving as many Inte.G.R.O meetings are dedicated to Problem-Solving Training, focusing on personal problems related to the life of patients. In our opinion this improvement may also be associated to the cognitive flexibility as already shown in the first two studies at the end of active treatment. In fact, on the M-FPT, post hoc Wilcoxon Signed-Ranks tests indicated that there was a significant difference in the t1 vs t0 score. We would like to pinpoint that several meetings could influence the flexibility such as “Jumping to conclusion”, “understanding the other’s mind”, “Self-control of anger” and “calm your mind”. Of course, the role of problem solving is of great importance. We hypothesize that its effect is two-fold, one directly on the cognitive functions and another one on personal and social functioning after a specific training in solving practical, interpersonal and personal problems. This putative two-fold effect on the cognition and social functioning should be better investigated. Falloon [35], on the basis of his studies and experiences in real world, affirmed that “problem solving training may be associated with substantial clinical and social benefits for people with major mental disorders”.. In addition, in Italy we had two important researches about good cognitive and social functioning outcomes after Problem Solving Training that support our hypothesis. In the first study by Barbieri et al. (2006) “after completion of the problem-solving program, significant improvements were noted in symptom scores on the Positive and Negative Syndrome Scale and in problem-solving and neurocognitive test performance, with further improvements six months after completion of the group sessions.” . In the second research by Veltro et al. [22] results showed that the Problem Solving training was effective in psychopathological measures and in social functioning, and also improved capacities for planning and memory. The interaction of cognition, life and social functioning in this field is also sustained by the recent experimental theory by Sarathy about the Real World Problem Solving [36]. In conclusion working with Problem Solving applied to practical, interpersonal and personal problems in real life could be successful. We also found an improvement in the level of stress (*p* < 0.01) at 3 year follow-up vs 1 year follow-up. This is relevant according to the underlying theory of stress-vulnerability model. According to the recent scientific literature [37] the mindfulness techniques, used in our approach within the meetings of “calm your mind” and related booster sessions, are relevant to stress-management. However, we would also emphasize that this secondary outcome should be also related to the similar improvement observed in cognitive flexibility associated in some research with resilience to negative life events and stress in adulthood [38]. We can confirm what sustained in the previous study, that “the process of salience detection, partially impaired in people with psychosis, is the first step towards attention and subsequent implementation of flexible responses” [6]. Even in this case the role of Problem Solving should be considered of primary importance. The good results observed in facing stress are very relevant for real patients’ life.. Since the ‘90s there is an increasing awareness that stress is ubiquitous, [39, 40] according to the concept of daily challenges from Antonovsky’s theory [8]. In fact, stress arises from every practical problem we have to deal with as well as from interpersonal relationships (real or virtual). Bearing this in mind, the approach focused on Personal Goals, Problem Solving, Stress Management with Life-Skills Training is welcome for dealing with “daily challenges”. This kind of intervention integrates the principles of salutogenic Antonovsky’s theory with Falloon’s [11] statement that Problem Solving training aims at enhancing the patient’s ability to “act wisely in facing practical problems as well as in social and interpersonal encounters by learning to consider the point of view of others and appraise the problems that must be addressed in an accurate manner”. In addition it has much in common with well-known social problem solving and cognitive enhancement therapies [30, 31, 41], even if our approach differs under many aspects, including social cognition, mindfulness, emotional literacy, communication skills and specific coping strategies.

It is difficult to compare the Inte.G.R.O approach with other kinds of approach such as E-IMR; these difficulties arise both from the limited number of studies about Inte.G.R.O and from differences in the methodology of outcome studies. Our data cannot clarify if the attention shift from illness management to the Problem-Solving applied to practical, interpersonal and personal problems can lead to differences in the outcome of the two interventions. It would be therefore useful to test the effects of Inte.G.R.O and E-IMR on dimensions relevant to recovery in a head-to head comparison design. The E-IMR remains the most studied approach, even if more research is needed for E-IMR to get a solid answer on its effectiveness on social functioning [4].

## Conclusion

The purpose of this study was to assess long-term real-life improvements in key recovery variables after an innovative psychoeducational and salutogenic program conceived to facilitate recovery. This approach is structured, manualized and it supports a recovery path for people with severe mental disorders, based on a salutogenic approach rather than a “reparative” one. In this context, we consider self-determination a cornerstone of recovery, and believe the ability to choose personal goals, the methods to reach them and the providers promoting them should all be essential components of a mental health service [42]. Overall, we consider these results very encouraging. Beyond being effective, this program may also be efficient, considering that it consists of a limited number of sessions and that the improvement is preserved and consolidates over time. Further studies, with larger samples and randomized controlled design, in several contexts and different geographic areas, are needed to prove the efficacy of Inte.G.R.O.

## Supplementary Information

**Additional file 1.**

## Data Availability

The datasets generated and/or analysed during the current study are not publicly available because data are currently under analysis for further pubblication but are available from the corresponding author on reasonable request.
