# Supplementary material for: Long term outcome study of a salutogenic psychoeducational recovery oriented intervention (Inte.G.R.O.) in severe mental illness patients
Source: BMC Psychiatry. 2022 Apr 5;22:240. doi: 10.1186/s12888-022-03887-2 (PMC8981821; doi:10.1186/s12888-022-03887-2)
Supplement: Supplementary file 1 — Additional file 1. [file 12888_2022_3887_MOESM1_ESM.docx]

Appendix 1

InteGRO Meetings (IM).

| IM1 | Presentation of the Intervention |
| --- | --- |
| IM 2 | Defining a Pleasant Goal |
| IM 3-5 | Emotional literacy: the Joyful, the Sadness, the Fear |
| IM 6-7 | Problem solving for Practical Problems: Teaching session, Training Session |
| IM 8-9 | Communication Skills: Expressing pleasant feelings and make positive request,  Active listening |
| IM 10 | Emotional literacy: the Shame |
| IM 11 | Problem Solving for Interpersonal Problems: expanding the social network |
| IM 12 | Defining a Personal Goal |
| IM 13 | Jumping to conclusions |
| IM 14 | Review Meeting |
| IM 15 | Emotional literacy: the Anger |
| IM 16-17 | Understanding your mind: training on connecting one's emotions-cognitions-behaviors for an event that occurred during the previous week |
| IM 18-20 | Understanding the other’s mind: training on predicting connections of other's emotions-cognitions-behaviors for an event that occurred during the previous week |
| IM 21 | Understanding the other’s mind: recognition of the Anger. |
| IM 22 | Communication Skills: Expressing unpleasant feelings |
| IM 23 | Training about Self-control of anger and aggressiveness |
| IM 24-25 | Calm your mind: breathing with awareness |
| IM 26 | Review Meeting |
| IM 27 | Effective communication and conversation |
| IM 28-32 | Problem Solving for Personal Problems: Problem Analysis, How to deal with a personal problem, How to deal with an emotional crisis, I wish to meet new people, How to improve physical wellbeing |
| IM 33-36 | 4 Biweekly Booster Session with use of Problem Solving |
|  | 3 Monthly Booster Session with use of Problem Solving |
|  | 3 Three-Monthly Booster Session with use of Problem Solving. |
